# Validation of Age-adjusted Shock indices for Predicting In-hospital outcomes in percutaneously REvascularized ST-elevation myocardial infarction - ASPIRE-STEMI study

**DOI:** 10.1016/j.ihj.2025.10.004

**Published:** 2025-10-10

**Authors:** Bryan Jacob Koithara, Ravi Kalra, Shashikala Sangle, Supriya Barsode, Shrikant Deshmukh

**Affiliations:** a Department of General Medicine, Bharati Vidyapeeth (Deemed to be University) Medical College and Hospital, Pune-Satara Road, Dhankawadi, Pune, Maharashtra, 411043, India; b Department of Cardiology, Bharati Vidyapeeth (Deemed to be University) Medical College and Hospital, Pune-Satara Road, Dhankawadi, Pune, Maharashtra, 411043, India; c Department of CRPU, Bharati Vidyapeeth (Deemed to be University) Medical College and Hospital, Pune-Satara Road, Dhankawadi, Pune, Maharashtra, 411043, India

**Keywords:** ST-Elevation myocardial infarction, Percutaneous coronary intervention, Risk scores, Age-modified shock index, Age-shock index, GRACE score, Major adverse cardiac events

## Abstract

The ASPIRE-STEMI study prospectively evaluated 236 patients with ST-elevation myocardial infarction undergoing percutaneous revascularization to validate Age-Shock Index (Age-SI) and Age-Modified Shock Index (Age-MSI) as alternatives to the GRACE score for predicting in-hospital major adverse cardiovascular events (MACE) and all-cause mortality. For MACE (*n* = 60), optimal cut-offs yielded sensitivities/specificities of 76.7 %/67 % (Age-SI ≥ 36.95), 85 %/56.2 % (Age-MSI ≥45.64), and 60 %/81.9 % (GRACE ≥127.5). For all-cause mortality (*n* = 17), optimal cut-offs yielded sensitivity/specificity of 82.4 %/83 % (Age-SI ≥46.83), 77 %/89 % (Age-MSI ≥67.35), and 94 %/76.7 % (GRACE ≥127.5). While each index independently predicted in-hospital outcomes, Age-SI and Age-MSI offer simple, bedside risk stratification in Indian STEMI patients post-PCI.

## 1 Introduction

Risk stratification after ST-elevation myocardial infarction (STEMI) is critical for guiding prognostication and patient care.^1^ The Global Registry of Acute Coronary Events (GRACE) score is widely used and validated but employs a complex algorithm that utilizes vital parameters, laboratory data and the Killip classification system,^2^ limiting its accessibility in resource-constrained or emergency settings. Simple age-adjusted indices derived from vital measurements, like the Age-SI and Age-MSI, have been proposed to improve bedside applicability. However, their validation has largely been limited to non-Indian retrospective registry-based cohorts, with most studies examining outcomes after primary PCI,^3^^,^^4^ fibrinolysis, or conservative therapy.^5^ There are no contemporary studies evaluating their prognostic potential for in-hospital outcomes after PCI under the pharmaco-invasive strategy. In India, where pharmaco-invasive strategies remain common^6^^,^^7^ and timely laboratory access may be variable, practical bedside tools are particularly valuable. We therefore conducted the ASPIRE-STEMI study to evaluate the prognostic performance of Age-SI and Age-MSI for predicting in-hospital MACE and all-cause mortality.

## 2 Methods

**Fig. 1:**
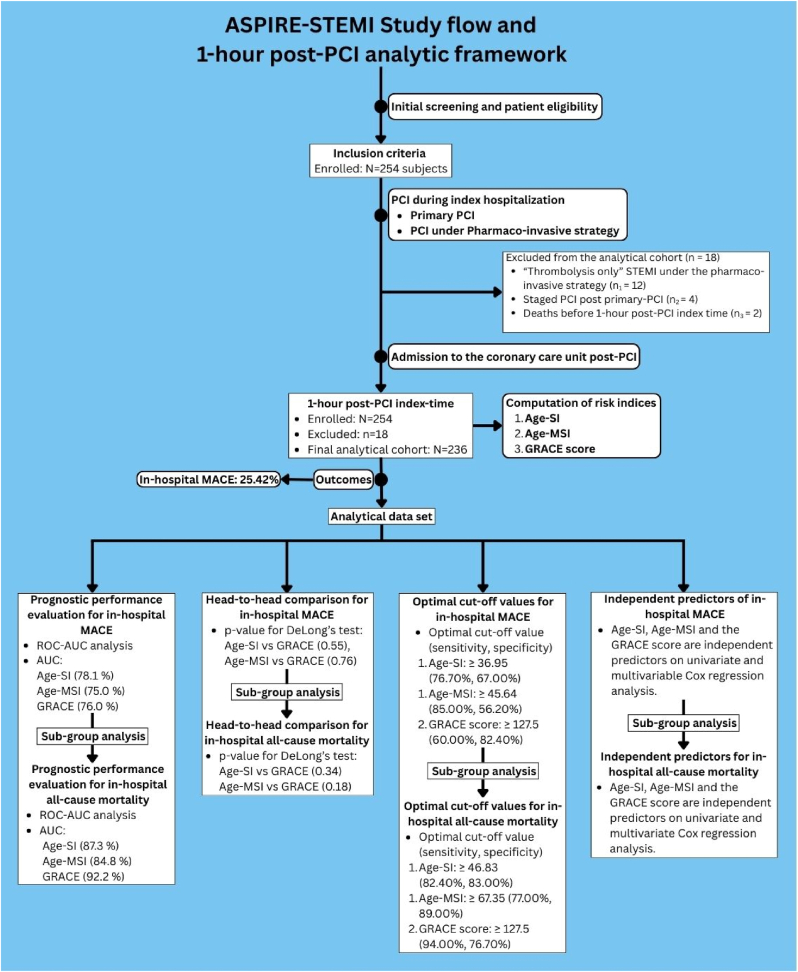
Central illustration showing the overview of the ASPIRE-STEMI study flow and 1-h post-PCI analytical framework for prognostic performance evaluation and validation of Age-SI and Age-MSI, and their comparison to the GRACE score (STEMI: ST-elevation myocardial infarction; PCI: percutaneous coronary intervention; Age-SI: Age-shock index; Age-MSI: Age-modified shock index; GRACE: Global Registry of Acute Coronary Events; MACE: major adverse cardiovascular events; ROC-AUC: Receiver Operating Characteristic-Area Under the Curve).

This prospective, observational, single-centre study was conducted from August 2022 to January 2024, following approval by our Institutional Ethics Committee (BVDUMC/IEC/09) and in accordance with the principles of the Declaration of Helsinki. Written informed consent was obtained from all participants. Study inclusion criteria included all patients aged 18 years and older with a primary diagnosis of STEMI presenting within 12 h of symptom onset, planned for primary-PCI or PCI under the pharmaco-invasive strategy as per current recommendations,^1^ and consenting to participate in the study (*n* = 254). Study exclusion criteria included STEMI patients with structural heart disease, congenital heart disease and pregnant females. All enrolled patients underwent clinical and demographic evaluations, routine blood tests, electrocardiography, and 2D echocardiography. A diagnosis of STEMI was based on the Fourth Universal Definition of Myocardial Infarction.^8^ “Thrombolysis-only” STEMI patients under the pharmaco-invasive strategy (n_1_ = 12), Staged-PCI post primary-PCI (n_2_ = 4), and deaths occurring before the 1-h post-PCI index time (n_3_ = 2) were excluded from the final analytical cohort (*n* = 236). Intra-aortic balloon pump (IABP) was used only as a standby or temporary hemodynamic support during PCI, when indicated, and heart rate and blood pressure were measured non-invasively in the resting state at 1-h post-PCI index time. From these values, age-adjusted shock indices were calculated: Age-SI and Age-MSI. The GRACE score was also derived at the same index time using the online calculator. All patients received standard post-PCI guideline-directed medical therapy, including dual antiplatelet therapy (aspirin and a P2Y12 inhibitor), high-intensity statins, beta-blockers, and renin–angiotensin system inhibitors/angiotensin receptor-neprilysin inhibitors as tolerated. A central illustration showing the overview of the ASPIRE-STEMI study flow and 1-h post-PCI analytical framework is given in Fig. 1.

The primary study outcome was in-hospital MACE, defined as a 6-point composite of acute heart failure,^9^^,^^10^ cardiogenic shock,^11^ malignant arrhythmia,^12^ coronary procedure-related myocardial infarction,^8^ stroke,^13^ and all-cause mortality. Criteria and definitions of study inclusions, exclusions, age-adjusted shock indices and outcomes are provided in the Supplementary Material – Operational criteria and definitions. Primary biostatistical analysis was performed for the MACE outcome, followed by subgroup analysis specifically for the outcome of all-cause mortality. Predictive performance of each score was assessed using Receiver Operating Characteristic (ROC) curve analysis, with optimal cut-offs determined by Youden's Index, and a head-to-head comparison between indices by DeLong's test. Sensitivity, specificity, odds ratios, and 95 % confidence intervals were reported. Univariate and multivariate logistic regression were used to identify independent predictors of MACE outcome on primary analysis, and separately for all-cause mortality on subgroup analysis. Statistical significance was set at *p* < 0.05.

## 3 Results

### 3.1 Population characteristics

Of 236 STEMI patients in the final analytical data set, in-hospital MACE occurred in 60 (25.4 %). Patients who developed MACE had a higher mean Age-SI (48.80 ± 16.99 vs. 33.88 ± 10.05), Age-MSI (63.93 ± 23.60 vs. 45.75 ± 14.24) and GRACE score (133.08 ± 32.31 vs. 103.03 ± 24.81). Patients with MACE were older (61.22 ± 13.35 vs. 56.47 ± 12.91). A higher proportion of females developed MACE (40 % vs. 27.8 %) compared to males (60 % vs. 72.6 %). Surprisingly, comorbidities like hypertension (45.00 % vs. 47.73 %), diabetes (41.46 % vs. 41.48 %), and a prior history of myocardial infarction (18.18 % vs. 6.67 %) were marginally less prevalent in those who developed MACE. Serum creatinine levels, however, were recorded as higher in the MACE group (1.22 ± 0.51 vs. 0.96 ± 0.34).

The most common type of STEMI in the MACE group was anterior wall (56.67 % vs. 53.14 %), followed by inferior wall (38.33 % vs. 40 %). 2D echocardiography showed a greater proportion of patients with an ejection fraction less than 25 % in the MACE group (6.67 % vs. 0.57 %). A higher prevalence of single vessel involvement (55.00 % vs. 52.27 %) and left anterior descending culprit artery involvement (78.83 % vs. 74.43 %) was also observed in the MACE group. Most patients in the MACE group received PCI under the pharmaco-invasive strategy (33.33 % vs. 27.84 %), while those who did not predominantly underwent primary-PCI (72.15 % vs. 66.66 %). Finally, the MACE group had a mean duration of hospital stay compared to those who did not (5.25 ± 7.31 vs. 3.34 ± 1.31). The demographic, clinical and biochemical characteristics of the participants are presented in Table 1.

**Table 1:** Baseline demographic, clinical and biochemical characteristics of the study population.

Variable	In-hospital MACE		
	No MACE (*n* = 176)	Developed MACE (*n* = 60)	*p-*value
GRACE score	103.03 (±24.81)	133.08 (±32.31)	<0.001
Age–SI	33.88 (±10.05)	48.80 (±16.99)	<0.001
Age–MSI	45.75 (±14.24)	63.93 (±23.60)	<0.001

Age (years)	56.47 (±12.91)	61.22 (±13.35)	0.02
Male gender	127 (72.16)	36 (60.00)	0.08
Female gender	49 (27.84)	24 (40.00)	0.08
Hypertension	84 (47.73)	27 (45.00)	0.72
Diabetes	73 (41.48)	25 (41.46)	0.10
Prior MI	32 (18.18)	4 (6.67)	0.03
Hemoglobin (g/dL)	13.17 (±2.16)	12.72 (±1.97)	0.16
Creatinine (mg/dL)	0.96 (±0.34)	1.22 (±0.51)	<0.001
Anterior wall STEMI	93 (53.14)	34 (56.67)	0.83
Inferior wall STEMI	70 (40.00)	23 (38.33)	0.83
Other STEMI	13 (6.86)	3 (5.00)	0.83
**Ejection fraction**			
>50 %	52 (29.55)	12 (20.00)	0.01
40–49 %	97 (55.11)	31 (51.67)	0.01
25–39 %	26 (14.77)	13 (21.67)	0.01
<25 %	1 (0.57)	4 (6.67)	0.01
Single vessel disease	92 (52.27)	33 (55.00)	0.72
Double vessel disease	70 (39.77)	23 (38.33)	0.84
Triple vessel disease	14 (7.95)	4 (6.66)	0.75
Left anterior descending artery	131 (74.43)	47 (78.33)	0.54
Left circumflex artery	50 (28.57)	9 (15.00)	0.04
Right coronary artery	82 (46.59)	30 (50.00)	0.65
Primary-PCI	127 (72.15)	40 (66.66)	0.42
Pharmaco-invasive PCI	49 (27.84)	20 (33.33)	0.42
Duration of hospital stay (days)	3.34 (±1.31)	5.25 (±7.31)	<0.001

Continuous variables expressed as mean ± standard deviation; discrete variables expressed as n (%).

MI: Myocardial infarction; STEMI: ST-elevation myocardial infarction; PCI: percutaneous coronary intervention; GRACE: Global Registry of Acute Coronary Events.

### 3.2 In-hospital MACE (primary analysis)

ROC analysis (Supplementary Material – ROC curves) demonstrated significant correlation of Age-SI, Age-MSI, and GRACE for the primary outcome of in-hospital MACE. The AUCs were 0.781 (95 % CI: 0.715–0.847) for Age-SI, 0.750 (95 % CI: 0.679–0.820) for Age-MSI, and 0.760 (95 % CI: 0.687–0.834) for GRACE (Supplementary Material – Tables; Table 1). Optimal cut-offs identified by Youden's Index were 36.95 (sensitivity - 76.7 %, specificity - 67 %) for Age-SI, 45.64 (sensitivity - 85 %, specificity - 56 %) for Age-MSI, and 127.5 (sensitivity - 60 %, specificity - 82.4 %) for the GRACE score (Supplementary Material – Tables; Table 1). Head-to-head comparison using DeLong's test (Age-SI vs. GRACE; *p*-value = 0.55, Age-MSI vs. GRACE; *p*-value = 0.576) showed no statistically significant differences between the AUCs (Supplementary Material – Tables; Table 2). On univariate Cox regression, each index was significantly associated with a higher risk of in-hospital MACE: Age-SI (OR 6.54, 95 % CI 3.33–12.86, *p* < 0.001), Age-MSI (OR 7.19, 95 % CI: 3.38–15.29, *p* < 0.001), and GRACE (OR 7.01, 95 % CI: 3.67–13.38, *p* < 0.001). After multivariate Cox regression analysis, all three indices were determined to be independent predictors of MACE: Age-SI (OR 4.42, 95 % CI: 1.96–9.98, *p* < 0.001), Age-MSI (OR 3.75, 95 % CI: 1.53–9.18, *p* = 0.004), and GRACE (OR 8.52, 95 % CI: 3.81–19.03, *p* < 0.001) (Supplementary Material – Tables; Table 3).

### 3.3 In-hospital all-cause mortality (subgroup analysis)

Seventeen patients (7.2 %) died during hospitalization. ROC analysis (Supplementary Material – ROC curves) demonstrated significant predictive value of all three indices. The AUCs were 0.873 (95 % CI: 0.771–0.974) for Age-SI, 0.848 (95 % CI: 0.734–0.961) for Age-MSI, and 0.922 (95 % CI: 0.869–0.975) for GRACE (Supplementary Material – Tables; Table 1). Optimal cut-offs identified by Youden's Index were 46.83 (sensitivity - 82.4 %, specificity - 83 %) for Age-SI, 67.35 (sensitivity - 77 %, specificity - 89 %) for Age-MSI, and 127.5 (sensitivity - 94.00 %, specificity - 76.7 %) for the GRACE score (Supplementary Material – Tables; Table 1). Head-to-head comparison by DeLong's test (Age-SI vs. GRACE; *p*-value = 0.34, Age-MSI vs. GRACE; *p*-value = 0.18) showed no statistically significant differences between the AUCs (Supplementary Material – Tables; Table 2). On univariate Cox regression, Age-SI (OR 22.95, 95 % CI: 6.28–83.89, p < 0.001), Age-MSI (OR 26.40, 95 % CI: 7.96–87.51, *p* < 0.001), and GRACE (OR 52.70, 95 % CI: 6.82–407.14, *p* < 0.001) were all significantly associated with mortality. After multivariate Cox regression analysis, each index remained an independent predictor: Age-SI (OR 21.68, 95 % CI: 3.05–153.73, *p* = 0.002), Age-MSI (OR 68.22, 95 % CI: 5.77–805.75, *p* < 0.001), and GRACE (OR 56.75, 95 % CI: 5.05–636.94, *p* = 0.001) (Supplementary Material – Tables; Table 3).

## 4 Discussion

The ASPIRE-STEMI study observed that Age-SI and Age-MSI demonstrated a favorable prognostic performance for in-hospital 6-point MACE [AUC - 0.82 (95 % CI: 0.76–0.88)] and all-cause mortality [AUC - 0.81 (95 % CI: 0.75–0.87)], comparable to the GRACE score on a head-to-head comparison of AUCs for MACE and all-cause mortality (*p* > 0.05). Youden's index analysis confirmed optimal cut-off values of Age-SI [36.95 (sensitivity - 76.7 %, specificity - 67 %), Age-MSI [45.64 (sensitivity - 85 %, specificity - 56 %), and the GRACE score [127.5 (sensitivity - 60 %, specificity - 82.4 %) for MACE. Likewise, optimal cut-offs for all-cause mortality were 46.83 (sensitivity - 82.4 %, specificity - 83 %), 67.35 (sensitivity - 77 %, specificity - 89 %), and the GRACE score [127.5 (sensitivity - 94.00 %, specificity - 76.7 %). The present study further demonstrated that both Age-SI and Age-MSI were independent predictors for in-hospital MACE and all-cause mortality on univariate and multivariate Cox regression analysis. Specifically concerning our study cohort, while Age-SI and Age-MSI did not detect additional instances of all-cause mortality compared with the GRACE score, they identified 9 and 14 additional MACE events, respectively, underscoring their utility in capturing broader in-hospital adverse outcomes. Our findings extend prior evidence on the prognostic utility of age-adjusted indices. Sadeghi et al^4^ identified an Age-SI ≥ 39.5 as the optimal threshold for 1-year MACE in Iranian STEMI (*n* = 818) patients receiving PCI, without a head-to-head comparison to contemporary risk indices. Similarly, in their analysis of the Henan registry (*n* = 3389), Wang et al^5^ determined Age-SI and Age-MSI with cut-offs of 51.1 and 46.6, respectively, as predictors of post-discharge mortality in STEMI post revascularization (PCI and thrombolysis), with discrimination comparable to TIMI and GRACE score. Zhou et al^3^ uniquely restricted analysis to 983 PCI-treated STEMI patients, reporting Age-SI (53.2, 47.53) and Age-MSI (64.47, 60.52) as predictors for in-hospital adverse events and mortality, respectively, with accuracy similar to GRACE, although vitals were recovered within a broad 24-h post-PCI window. Restricting outcomes to PCI-treated patients offers the advantage of relatively standardized care pathways, whereas thrombolysis-only cohorts diverge into successful lysis (routine early angiography within 2–24 h) and failed lysis (immediate rescue-PCI), introducing heterogeneity that may dilute prognostic signals. To the best of our knowledge, the ASPIRE-STEMI study is the first to evaluate Age-SI and Age-MSI prospectively, in a restricted cohort of STEMI patients undergoing PCI (both primary and pharmaco-invasive), with indices calculated at a standardised 1-h post-PCI index time. This approach minimizes variability and bias. Importantly, the performance of Age-SI and Age-MSI in our study was not statistically different from GRACE despite requiring only bedside vital measurements and age. This has particular relevance in Indian settings, where pharmaco-invasive PCI remains common and access to timely laboratory data may be variable. The present study had several limitations, which included (a) single-centre study with a modest sample size, limiting external validation, (b) not assessing dynamic changes of indices after the 1-h post-PCI index time, (c) not assessing longer-term prognostic utility, (d) potential recruitment bias arising out of limiting our study to STEMI undergoing PCI and (d) not capturing detailed post-PCI pharmacotherapy data. The ASPIRE-STEMI study recognizes Age-SI and Age-MSI as rapid bedside tools for early risk stratification in a small subgroup of Southeast Asian PCI-treated STEMI patients as an alternative to established scores such as the GRACE score. However, given the limitations of the present study, further large prospective multi-center cohort studies are needed to further evaluate the prognostic role of Age-SI and Age-MSI. Their simplicity may facilitate decision-making in resource-constrained environments, aiding triage and intensity of monitoring during hospitalization.

## Author contribution

All authors equally contributed to the study and manuscript development and finalisation.

## Funding

This research did not receive any specific grant from funding agencies in the public, commercial, or not-for-profit sectors.

## Declaration of competing interest

The authors declare that they have no known competing financial interests or personal relationships that could have appeared to influence the work reported in this paper.
