# Supplementary material for: Validation of Age-adjusted Shock indices for Predicting In-hospital outcomes in percutaneously REvascularized ST-elevation myocardial infarction - ASPIRE-STEMI study
Source: Indian Heart J. 2025 Oct 10;77(6):462–6. doi: 10.1016/j.ihj.2025.10.004 (PMC12793910; doi:10.1016/j.ihj.2025.10.004)
Supplement: Multimedia component 1 [file mmc1.docx]

Supplementary Material – Operational criteria and definitions

1. Study inclusion
   1. STEMI was defined and diagnosed as per the European Society of Cardiology/American College of Cardiology Foundation/American Heart Association/World Heart Federation Task Force for the Fourth Universal Definition of Myocardial Infarction.
   2. Primary-PCI is defined as an emergent percutaneous catheter intervention in the setting of STEMI, without previous fibrinolytic therapy treatment within 12 hours from symptom onset, but even later if symptoms and ST-segment elevation are persisting or evolving.
   3. PCI under the pharmaco-invasive strategy, defined as STEMI treated with fibrinolytic therapy followed by coronary angiography within 3 to 24 h with the intent to perform PCI.
2. Study exclusions
   1. Structural heart disease
   2. Congenital heart disease
   3. Pregnancy
3. Exclusions from the final analytical cohort
   1. “Thrombosis-only” STEMI under the pharmaco-invasive strategy is defined as STEMI patients who initially consented to undergo PCI under the pharmaco-invasive strategy but subsequently withdrew consent after initial fibrinolytic therapy.
   2. Staged-PCI post primary-PCI are defined as selected hemodynamically stable patients with STEMI and multivessel disease undergoing staged PCI of a significant non-infarct artery stenosis after primary PCI.
   3. Deaths occurring before the 1-hour post-PCI index time are defined as deaths, including periprocedural deaths, occurring before the 1-hour post-PCI index time when vital measurements are undertaken to compute risk indices under evaluation.
4. Formulae for age-adjusted shock indices
5. Age-SI = age × (heart rate ÷ systolic blood pressure)
6. Age-MSI = age × (heart rate ÷ mean arterial pressure)
7. In-hospital MACE 6-point composite outcome
8. Acute heart failure is defined as the rapid onset or worsening of symptoms and signs of heart failure, characterized by congestion and/or hypoperfusion, requiring urgent therapy, typically with intravenous medications. The term encompasses acute de novo presentations as well as acute decompensation of chronic heart failure.
9. Cardiogenic shock is defined as systolic blood pressure <90 mm Hg for >30 min or the need for supportive management to maintain systolic blood pressure >90 mm Hg; clinical signs of pulmonary congestion; and evidence of impaired end-organ perfusion with at least 1 of the following: cool extremities, decreased urine output, increased lactic acid level, or altered mental status
10. Malignant arrhythmias are defined as either ventricular tachycardia (VT) or ventricular fibrillation (VF) or bradyarrhythmias such as atrioventricular block causing hemodynamic compromise or cardiac arrest.
11. Coronary procedure-related myocardial infarction was defined and diagnosed as per the European Society of Cardiology/American College of Cardiology Foundation/American Heart Association/World Heart Federation Task Force for the Fourth Universal Definition of Myocardial Infarction for (a) PCI-Related Myocardial infarction ≤ 48 hours after the index procedure (Type 4a), (b) stent/scaffold thrombosis associated with PCI (Type 4b), and restenosis associated with PCI (Type 4c).
12. Stroke is defined as a neurological deficit that persists for longer than 24 hours attributed to an acute focal injury of the central nervous system by an ischemic or non-ischemic vascular cause.
13. All-cause mortality – Defined as death occurring during index hospitalization due to any cause related to the indication for index hospitalization.
