# Supplementary material for: Validation of Age-adjusted Shock indices for Predicting In-hospital outcomes in percutaneously REvascularized ST-elevation myocardial infarction - ASPIRE-STEMI study
Source: Indian Heart J. 2025 Oct 10;77(6):462–6. doi: 10.1016/j.ihj.2025.10.004 (PMC12793910; doi:10.1016/j.ihj.2025.10.004)
Supplement: Multimedia component 2 [file mmc2.docx]

Supplementary Material – ROC curves


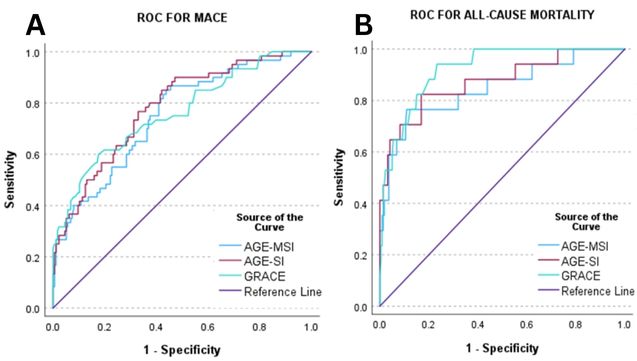


Caption – Receiver operating characteristic (ROC) curves for GRACE score, Age-SI, and Age-MSI for STEMI patients. (A) ROC curve for in-hospital MACE; (B) ROC curve for all-cause mortality. AGE-SI: Age-shock index; AGE-MSI: Age-modified shock index.
