# Supplementary material for: Validation of Age-adjusted Shock indices for Predicting In-hospital outcomes in percutaneously REvascularized ST-elevation myocardial infarction - ASPIRE-STEMI study
Source: Indian Heart J. 2025 Oct 10;77(6):462–6. doi: 10.1016/j.ihj.2025.10.004 (PMC12793910; doi:10.1016/j.ihj.2025.10.004)
Supplement: Multimedia component 3 [file mmc3.docx]

Supplementary Material – Tables

| **Table 1. Prognostic performance of Age-SI, Age-MSI, and the GRACE score** | | | | | | |
| --- | --- | --- | --- | --- | --- | --- |
| **Outcomes** | **Cut-off** | **AUC** | **95% CI** | **Sensitivity** | **Specificity** | ***p-value*** |
| **In-hospital MACE** | | | | | | |
| Age-SI | 36.95 | 0.781 | 0.715 – 0.847 | 76.70% | 67.00% | <0.001 |
| Age-MSI | 45.64 | 0.750 | 0.679 – 0.820 | 85.00% | 56.20% | <0.001 |
| GRACE score | 127.5 | 0.760 | 0.687 – 0.834 | 60.00% | 82.40% | <0.001 |
| **In-hospital all-cause mortality** | | | | | | |
| GRACE score | 127.5 | 0.922 | 0.869 – 0.975 | 94.00% | 76.70% | <0.001 |
| Age-SI | 46.83 | 0.873 | 0.771 – 0.974 | 82.40% | 83.00% | <0.001 |
| Age-MSI | 67.35 | 0.848 | 0.734 – 0.961 | 77.00% | 89.00% | <0.001 |
| AUC: Area under curve; CI: confidence intervals; GRACE: Global Registry of Acute Coronary Events | | | | | | |

| **Table 2. Comparing the prognostic performances of age-SI, age-MSI and the GRACE score** | | |
| --- | --- | --- |
| **Clinical outcomes** | **AUC difference (95% CI)** | ***p* - value** |
| **Inpatient MACE** | | |
| Age-SI versus GRACE score | -0.021 (-0.0479 - 0.0890) | 0.555 |
| Age-MSI versus GRACE score | -0.01 (-0.0794 - 0.0580) | 0.760 |
| **Inpatient all-cause mortality** |  |  |
| Age-SI versus GRACE score | -0.049 (-0.151 - 0.053) | 0.346 |
| Age-MSI versus GRACE score | -0.074 (-0.184 - 0.036) | 0.188 |
| AUC: area under the curve; CI: confidence interval; GRACE: Global Registry of Acute Coronary Events | | |

| **Table 3. Effects of multiple variables on Age-SI, Age-MSI and GRACE score on in-hospital MACE and all-cause mortality on univariate and multivariate Cox regression analysis** | | | | | |
| --- | --- | --- | --- | --- | --- |
| **Risk indices** | **Model** | **In-hospital MACE** | | **In-hospital all-cause mortality** | |
|  |  | **OR (95% CI)** | ***p* - value** | **OR (95% CI)** | ***p* - value** |
| Age-SI | Model 1 | 6.54 (3.33 - 12.86) | <0.001 | 22.95 (6.28 - 83.89) | <0.001 |
|  | Model 2 | 4.42 (1.96 - 9.98) | <0.001 | 21.68 (3.05 - 153.73) | 0.002 |
| Age-MSI | Model 1 | 7.19 (3.38 - 15.29) | <0.001 | 26.40 (7.96 - 87.51) | <0.001 |
|  | Model 2 | 3.751 (1.532- 9.184) | 0.004 | 68.22 (5.77 - 805.75) | <0.001 |
| GRACE score | Model 1 | 7.01 (3.67 - 13.38) | <0.001 | 52.70 (6.82 - 407.14) | <0.001 |
|  | Model 2^a^ | 8.523 (3.817- 19.029) | <0.001 | 56.75 (5.05 - 636.94) | 0.001 |
| OR: odds ratio; CI: confidence interval   - In model 1, univariate (unadjusted) analysis of age-SI, age-MSI and the GRACE scores as binary categorical variables according to the cutoff points determined by Youden’s index. - In model 2, a multivariate analysis of model 1 adjusted for gender, hypertension, diabetes, current tobacco use, history of myocardial infarction, hemoglobin, creatinine, anterior wall STEMI, inferior wall STEMI, ejection fraction, left anterior descending artery, right coronary artery, and left circumflex culprit artery, primary-PCI, PCI under the pharmaco-invasive strategy, and duration of hospital stay was performed. The GRACE score was not adjusted for serum creatinine in model 2^a^. | | | | | |
